# Recent Progress in Development of Tnt1 Functional Genomics Platform for *Medicago truncatula* and *Lotus japonicus* in Bulgaria

**DOI:** 10.2174/138920211795564313

**Published:** 2011-04

**Authors:** Miglena Revalska, Valya Vassileva, Sofie Goormachtig, Tom Van Hautegem, Pascal Ratet, Anelia Iantcheva

**Affiliations:** 1 AgroBioInstitute, Bul. Dragan Tzankov 8, Sofia 1164, Bulgaria; 2 Institute of Plant Physiology and Genetics, Acad. Georgi Bonchev Str., Bl. 21, 1113 Sofia, Bulgaria; 3 Department of Plant Systems Biology, VIB/Ghent University, Technologiepark 927, 9052 Ghent, Belgium; 4 ISV-CNRS, 1 Avenue de la Terrasse 91198, Gif-sur-Yvette Cedex, France

**Keywords:** Insertional mutagenesis, legume genomics, *Medicago truncatula*, *Lotus japonicus*, phenotyping, *Tnt1* mutants.

## Abstract

Legumes, as protein-rich crops, are widely used for human food, animal feed and vegetable oil production. Over the past decade, two legume species, *Medicago truncatula *and* Lotus japonicus,* have been adopted as model legumes for genomics and physiological studies. The tobacco transposable element, *Tnt1*, is a powerful tool for insertional mutagenesis and gene inactivation in plants. A large collection of *Tnt1*-tagged lines of *M. truncatula* cv. Jemalong was generated during the course of the project ‘GLIP’: Grain Legumes Integrated Project, funded by the European Union (www.eugrainlegumes.org). In the project ‘IFCOSMO’: Integrated Functional and COmparative genomics Studies on the MOdel Legumes *Medicago truncatula* and *Lotus japonicus*, supported by a grant from the Ministry of Education, Youth and Science, Bulgaria, these lines are used for development of functional genomics platform of legumes in Bulgaria. This review presents recent advances in the evaluation of the *M. truncatula Tnt1* mutant collection and outlines the steps that are taken in using the *Tnt1-*tagging for generation of a mutant collection of the second model legume *L. japonicus*. Both collections will provide a number of legume-specific mutants and serve as a resource for functional and comparative genomics research on legumes. Genomics technologies are expected to advance genetics and breeding of important legume crops (pea, faba bean, alfalfa and clover) in Bulgaria and worldwide.

## 1. PLANT GENOMIC AND POST-GENOMIC ERA WORLDWIDE AND IN BULGARIA

Plant genomics research started with a complete genome sequencing of the model plant *Arabidopsis thaliana* in December 2000 [1]. The Arabidopsis community proposed an ambitious goal to identify the function of every gene by 2010. Since then, an extremely rapid progress has been made in the field of plant genomics. An important step ahead was the publication of the rice genome sequence in 2002 [2, 3], draft genome of poplar in 2006 [4], whole genome sequence of two grapevine genotypes in 2007 [5], draft sequence of the genomes of *Lotus japonicus* [6, 7] and *Glycine max* in 2010 [8]. Currently, the genomes of several other plant species as barley, wheat, potato, cotton, tomato, maize, *Medicago truncatula* have also been extensively explored. The rapid accumulation of sequencing information and global gene expression analyses enable us to generate hypotheses about gene function and answer specific biological questions. A great challenge for the scientists of the post-genomic era is to analyze and understand function of every gene for all the sequenced genomes.

Legumes are protein-rich crop plants, used for human nutrition, animal feed and production of vegetable oil. A key contribution of legumes to sustainable agriculture and nitrogen cycle comes from their ability to fix atmospheric nitrogen in most agricultural ecosystems. They are able to form specialized symbiotic organs, the root nodules, in which rhizobial bacteria reside and reduce atmospheric nitrogen to ammonia. This process largely contributes to the nitrogen nutrition of the host plant, reducing the need for nitrogen fertilizers. Intensive application of chemical fertilizers has allowed developing and maintaining a very productive agriculture. However, this application has resulted in nitrogen leakage from agricultural systems into groundwater, rivers, coastal waters.

Over the past decade, two model legumes, *M. truncatula *[9, 10,] and *L. japonicus* [11-13] have been proposed for molecular genetics research. It is generally accepted that knowledge about certain shared characteristics of legumes, such as the pathways involved in symbiosis with rhizobia and synthesis of flavonoids and glycosides, is easily transferable from model plants to crops [14]. Thus, information from these two models would be useful for improvement of forage legumes, e.g. alfalfa and clovers, even for the study of agronomic traits (yield and growth habits), because of the close relation of *L. japonicus* and *M. truncatula* to the forage legume species *Lotus corniculatus* (birdsfoot trefoil) and *Medicago sativa* (alfalfa), respectively. Model legumes can also provide useful information and contribute to the improvement of other crops, such as tomato, sunflower, cotton, corn and rice. In addition, the nitrogen fixation process in legumes has a unique biochemistry [15] and many valuable molecules with an important biomedical application, such as isoflavones and plant sterols, are found among legume metabolites [16].

The ‘*Medicago truncatula*’ Sequencing Consortium is an international partnership of research laboratories, which is decoding the genome sequence of *M. truncatula*. It was founded by the Samuel Robert Noble Foundation and National Science Foundation in the USA, and by the European Union. The European Grain Legumes Integrated Project (GLIP) was launched in 2004 to increase knowledge in grain legume biology in order to develop new strategies for promoting the cultivation of legumes in Europe, as well as their use in animal feed. Legumes are a key component for the development of sustainable agriculture, but they remain underused in Europe. The ‘*Medicago truncatula*’ Sequencing Consortium has made significant contribution to the development of an expressed sequence tag (EST) resourse and genome sequencing. A collection of 268 712 ESTs that represents 38 238 unigenes and >89 Mb genomic sequences of the 200 Mb euchromatic regions have been made publicly available. Sequencing of the gene space of the eight Medicago chromosomes is almost completed (http://www.medicago.org).

In the frame of the project ‘IFCOSMO’: Integrated Functional and COmparative genomics Studies on the MOdel Legumes *Medicago truncatula* and *Lotus japonicus* (supported by a grant from the Ministry of Education, Youth and Science, Bulgaria), we investigate the utility and challenges of exploring forward and reverse genetic tools using the *Tnt1 *insertional mutant collection of *M. truncatula *created by AgroBioInstitute (ABI) during the GLIP project. In order to generate a mutant collection of the second model legume *L. japonicus*, particular attention is given to the use of the *Tnt1-*mediated**mutagenesis for establishment of starter lines for this model legume. Both collections will provide a number of legume-specific mutants and will serve as a base for advanced functional and comparative genomics research on the legumes.

## 2. *TNT1* AS A POWERFUL TOOL FOR INSERTIONAL MUTAGENESIS IN *MEDICAGO TRUNCATULA* AND MAYBE IN *LOTUS JAPONICUS*

Generation of large mutant collections and subsequent analysis of the effect of gene tagging and mutation represent a fundamental approach for understanding gene function and genetic interactions. Different tools are used for gene disruption and production of mutant collections. Establishment of several technological platforms, such as TILLING [17], microarrays of several thousand non-redundant *Lotus* cDNAs for transcriptomic and proteomic profiling [18,19], and advanced genome-sequencing programs [20] has made a major contribution to the functional genomics studies on the model legumes. A transposon-tagged *L. japonicus *mutant, *nin* (for nodule inception), has been isolated and characterized by Schauser *et al.* [21]. Imaizumi *et al*. [22] demonstrated that activation-tagging method can be successfully used in the model legume *L. japonicus. *Insertional mutagenesis *via *transposable elements [23, 24] represents attractive alternative for creation of large mutant collections. Class I mobile genetic elements, or retrotransposons, copy themselves and paste copies back into the genome at multiple places [25]. Initially retrotransposons copy themselves to RNA but, in addition to being transcribed, the RNA is copied into DNA by a reverse transcriptase [26] and is inserted back into the genome. Because there is no excision during replicative transposition, mutations generated by retrotransposon insertions are stable. Efficient transposition of the *Nicotiana tabacum* *Tnt1* retrotransposon in *M.* *truncatula* R108 line is firstly described by d’Erfurth *et al*. [27] during the early steps of *in vitro* transformation/regeneration process and confirmed for *M truncatula* cv. Jemalong 2HA [28]. The high efficiency of *Tnt1* transposition during the regeneration process results in lines, carrying multiple *Tnt1* inserts (from 4 to 20 insertions per regenerated plant) and most likely, resulting in the accumulation of multiple mutations. The *Tnt1* insertions are stable during the life cycle of *M. truncatula* and most of them are genetically independent and can be separated by recombination. In addition, *Tnt1 *seems to transpose preferentially into the gene rich regions of the *M. truncatula* genome [29, 30] and its multiplication by transposition can be re-induced by tissue culture. Several of the generated developmental and symbiotic *Tnt1*-tagged mutants have been already identified and characterised [31-33].

Based upon successful activity of the worldwide *Medicago *consortium, we propose an idea for initiation and development of a *Tnt1* mutant collection for the second model legume *L. japonicus*. This collection will provide a number of legume-specific mutants and will serve as a resource for advanced functional genomics research of *L. japonicus*. In addition, the prospect of integrating genome information from both model legumes to achieve a platform for comparative genomics will provide novel insights into the organization and evolution of legumes, as well as the similarities and differences with genomes of other plant families.

## 3. FUNCTIONAL LEGUME GENOMICS PLATFORM IN BULGARIA ON THE MOVE

Recent developments in the area of legume genomics provide exciting opportunities for transferring knowledge from models into crop legumes. To date, most progress in identifying genes important for many aspects of legume biology has been made in the two model legumes, *M. truncatula* and *L. japonicus*. In Bulgaria, *M. truncatula* research started with participation of ABI in the project ‘Molecular and cellular bases of somatic embryogenesis in alfalfa’, INCO-COPERNICUS (IC15-CT96-0906), FP5 of European Union. During the course of this study an efficient regeneration and transformation procedures for *M. truncatula* are established. Because of the successful project implementation, ABI was invited as a partner to the GLIP project (FP6, FOOD-CT-2004-506223). The main research task of ABI together with the other partners in work package (WP) 5.2 “*Tnt1* mutagenesis” was to develop a large-scale *Tnt1 *mutant collection of *M. truncatula*. The team contributed greatly in defining conditions for efficient *Tnt1* transposition, resulting in lines with a high number of inserts [28]. Seeds of the *M. truncatula* mutant lines are currently available at ABI, as well as at the stock centers for seed production in Szeged, Hungary and Dijon, France. Two of the partners of ABI in the WP 5.2 were the Institut des Sciences du Végétal, Centre national de la recherche scientifique (ISV, CNRS), represented by Dr. Pascal Ratet (the leader of WP 5.2), and the Department of Plant Systems Biology, VIB/University of Ghent represented by Prof. Sofie Goormachtig. Because of the common interest in further exploitation of the mutant lines generated during GLIP and common research activities, these two institutions have become partners in the IFCOSMO project. Another Bulgarian project partner is the Institute of Plant Physiology and Genetics (IPPG), Bulgarian Academy of Sciences, which provides expertise in Gateway cloning, hydroponic cultivation, and a number of imaging and biochemical techniques. Since 2002, *L. japonicus* has been used as a model plant for investigation of symbiotic relationships with *Mesorhizobium loti* or purified *M. loti* lipochitin oligosaccharide signal molecules (Nod factors). Visualization and quantification of cortical microtubules dynamics in living root hairs of *L. japonicus* have been achieved [34, 35].

## 4. RECENT PROGRESS IN IFCOSMO PROJECT

The *M. truncatula* tagged population is a very useful resource for investigation of gene function in legumes. In order to confirm the presence of new copies of the *Tnt1* retrotransposon, randomly selected lines from the Bulgarian *Tnt1* mutant collection were screened by a transposon display (TD) technique or Inverse PCR (IPCR). Thirty of the positive lines from T_1_ generation were selected for assessment of their phenotype characteristics and symbiotic properties, according to the visible morphological criteria for mutant evaluation [29]. The selected *Tnt1 *mutants from T_1_ and the following T_2_ generations were planted in a greenhouse and their root and shoot morphology, variations in plant growth, development, flowering and pigmentation were evaluated. After performing the greenhouse assessment, several phenotypic classes could be distinguished: the first and largest class comprises the lines with extreme and moderate dwarf phenotype (Supplemental Fig. (**1A**)); the second largest class possesses inflorescence and floral organ defects, yellow-greenish and variegated leaf colour, mixed foliage leaves, early or late flowering (Supplemental Fig. (**1B**)). One of the* Tnt1* lines showed quite unusual phenotypic features manifested by five- to seven-foliate leaves and defective flower development (Supplemental Fig. (**1C**)). Unfortunately, this line was sterile and did not produce any seeds.

Root morphology and development, and symbiotic characteristics of the mutant lines were evaluated *in vitro* on agar plates and *in vivo* in hydroponic systems. In view of the root architecture, three main phenotypic groups were identified: mutant plants with long and thin roots and reduced lateral branches represent the first group (Supplemental Fig. (**2A**)), the second group includes lines with short roots having many secondary branches (Supplemental Fig. (**2B**)), and the third group was presented by the lines with short and unbranched root architecture (Supplemental Fig. (**2C**)). Evaluation of the root development in T_1_ and T_2_ generations from seedling stage to maturity revealed mutant plants with very unusual root phenotype. These plants produce many secondary branches that grow parallel to each other on the main root, resembling a fish bone. Based upon this root structure, the mutant was named ‘fishbone’ (Supplemental Fig. (**2D**)). Sequencing of the *Tnt1* insertion sites (FSTs) for this line is in progress.

The selected *Tnt1* lines revealed a wide variability in their symbiotic characteristics*.* They could be grouped into several distinct classes: the most numerous group demonstrated complete absence of nodulation (Nod^-^), the second group formed white spherical nodule-like structures (Nod^+^, Fix^–^), the third class was characterized by a mixture of white (inefficient) and efficient nodules (Nod^+^Fix^–/+^), and very few lines formed an excessive number of nodules (Nod^++^). There were also *Tnt1* lines that could not be infected (Inf^-^), and a line that formed elongated efficient nodules (Supplemental Fig. (**3**)).

Part of the phenotyping activities on the selected *Tnt1 *mutants were focused on some aspects of leaf epidermal morphology [36]. The leaf epidermis is essential to plant functioning and survival not only because it is the first line of defense against environmental damages but also because of its crucial developmental role. Interactions between the epidermis and internal tissues regulate the overall leaf architecture. Selected *Tnt1 *insertion lines showed significant variations in leaf epidermal cell size, shape and the number and distribution of stomata. There were also visible variations in cell wall undulation, which was differently expressed in the upper and lower epidermis.

Pictures, microscope images and other data and information are collected in order to create an online catalogue of the investigated mutant lines.

As mentioned above, the plant genomic region that borders *Tnt1* (Flanking Sequence Tag, FST) can be identified by TD or Inverse PCR. All the FSTs that are sequenced by various partners are stored in a database for public use at the Samuel Roberts Noble Foundation (http://bioinfo4.noble.org/ mutant/). On the base of these sequences, two mutant lines (So 6142A И So 5945A) from the ABI’s collection were selected because of the location of their FSTs in the coding sequence of three important genes. They correspond to insertions in a 2OG-Fe (II) oxygenase (NP7260384, line 6142A, insertion 9), HAC1, transcription cofactor (NP7260991, line 5945A insertion 5) and Cyclin-like F-box protein (NP7270344, line 5945A insertion 7). Two FSTs partially correspond to the genes encoding an Auxin influx carrier protein, LAX3 (NP611743) and Transcriptional factor B3 family protein, similar to Auxin response factor (NP7272450). These five genes were selected for molecular cloning and characterization because of their involvement in fundamental processes of plant development, participation in secondary root branching, nitrogen fixation and somatic embryogenesis. The selected genes were cloned into overexpression and knockout vectors by the GATEWAY technology. Transcriptional reporters consisting of promoter fragments of the genes of interest, driving GFP and GUS, were also constructed. Building tools for studying transgenic *M. truncatula*, *L. japonicus* and *A. thaliana* (used as a reference species) plants, where these genes are either overexpressed or silenced through RNA interference (RNAi) technology [37] are currently in progress. In order to find homozygous ‘tag’ mutant individuals among F_1_ progeny of two of the above mentioned lines (insert 9 of line So 6142, and insert 5 and 7 of line So 5945), for the respective FSTs a reverse genetic approach will be used.

Assessment of experimental data for the generation of starter lines and *Tnt1* mutant collection for *M. truncatula* has shown the presence of the auxin 2.4-dichlorophenoxyacetic acid (2.4D) during callus tissue induction as an important component for successful transposition of the tobacco *Tnt1 *retroelement [28]. Besides the presence of 2.4D in culture media, other important components ensuring high transposition efficiency of *Tnt1* during regeneration of mutant lines are the induction of friable callus tissue to full dedifferentiation of plant explants, and the osmotic pretreatment of starter explants. Unfortunately, the efficiency of the existing *in vitro* techniques and procedures for regeneration and gene transfer in *L. japonicus* does not correspond to the requirement for effective* Tnt1* introduction and transposition. The data on the regeneration and gene transfer for *L. japonicus* published so far, illustrated successful use of hypocotyl explant and plant growth regulators like α-naphthaleneacetic acid (NAA) and 6-benzylaminopurine (BAP). Previously developed regeneration protocols induce compact callus tissue, followed by shoot development. One of the main objectives of the IFCOSMO project, concerning construction of *L. japonicus Tnt1* starter line and further generation of insertional mutant lines, is to develop an indirect somatic embryogenesis protocol using 2.4D as the main callus inducer. During the first stage of the project, new regeneration/transformation protocols were established, which allowed obtaining starter lines, carrying the *Tnt1* retrotansposon in a low copy number. The TD and IPCR analyses confirmed the presence of one to eight transposed copies of the element per line. The experimental data available for *M. truncatula* starter lines demonstrate that four to eight *Tnt1* copies are required for the newly introduced insertions [28]. The second part of the IFCOSMO project will focus on further evaluation of the starter lines and setting up conditions for efficient *Tnt1* transposition in *L. japonicus*.

Recent progress in molecular biology, genomics, transcriptomics, proteomics, metabolomics and bioinformatics for rice, *A. thaliana,* *M. truncatula* and *L. japonicus* has provided new opportunities for investigation of these model species [38-41]. The knowledge obtained by comparative genomics analyses of legumes and other model plants, such as Arabidopsis and rice, could allow the identification of legume specific systems and provide insights into biological phenomena of legume crops. Genomes of the model species *M. truncatula* and *L. japonicus* share considerable genetic synteny with crop legumes, which greatly facilitates gene discovery and understanding the complex relationships between genes and phenotypes. To support comparative genome analyses among legumes, up-to-date genetic and genomic databases, integrating information from multiple legume species have been developed (LIS, http://www.comparative-legumes.org/lis/ and LegumeDB, http://ccg.murdoch.edu.au/index).

Successful translation of accumulated knowledge from models to practical agriculture would lead to new discoveries about developmental processes and application of new strategies for crop improvement. In a long-term plan, development of functional and comparative genomics platform would be of particular agricultural significance, facilitating the genetics and breeding of important legume crops cultivated in Bulgaria and worldwide, such as pea, faba bean, alfalfa and clover.

## ABBREVIATIONS

ABI: = AgroBioInstitute
BAP: = 6-benzylaminopurine
EST: = expressed sequence tag
GLIP: = Grain Legumes Integrated Project
IFCOSMO: = Integrated Functional and COmparative genomics Studies on the MOdel Legumes
NAA: = α-naphthaleneacetic acid
TD: = transposon display
2.4D: = 2.4-dichlorophenoxyacetic acid

## References

[1] (2000). Arabidopsis Genome Initiative. Analysis of the genome sequence of the flowering plant *Arabidopsis thaliana*. Nature.

[2] Goff SA, Ricke D, Lan TH, Presting G, Wang R, Dunn M, Glazebrook J, Sessions A, Oeller P, Varma H, Hadley D, Hutchison D, Martin C, Katagiri F, Lange BM, Moughamer T, Xia Y, Budworth P, Zhong J, Miguel T, Paszkowski U, Zhang S, Colbert M, Sun WL, Chen L, Cooper B, Park S, Wood TC, Mao L, Quail P, Wing R, Dean R, Yu Y, Zharkikh A, Shen R, Sahasrabudhe S, Thomas A, Cannings R, Gutin A, Pruss D, Reid J, Tavtigian S, Mitchell J, El-dredge G, Scholl T, Miller RM, Bhatnagar S, Adey N, Rubano T, Tusneem N, Robinson R, Feldhaus J, Macalma T, Oliphant A, Briggs S (2002). A draft sequence of the rice genome (*Oryza sativa* L. ssp. japonica). Science.

[3] Yu J, Hu S, Wang J, Wong GKS, Li S, Liu B, Deng Y, Dai L, Zhou Y, Zhang X, Cao M, Liu J, Sun J, Tang J, Chen Y, Huang X, Lin W, Ye C, Tong W, Cong L, Geng J, Han Y, Li L, Li W, Hu G, Li J, Liu Z, Qi Q, Li T, Wang X, Lu H, Wu T, Zhu M, Ni P, Han H, Dong W, Ren X, Feng X, Cui P, Li X, Wang H, Xu X, Zhai W, Xu Z, Zhang J, He S, Xu J, Zhang K, Zheng X, Dong J, Zeng W, Tao L, Ye J, Tan J, Chen X, He J, Liu D, Tian W, Tian C, Xia H, Bao Q, Li G, Gao H, Cao T, Zhao W, Li P, Chen W, Zhang Y, Hu J, Liu S, Yang J, Zhang G, Xiong Y, Li Z, Mao L, Zhou C, Zhu Z, Chen R, Hao B, Zheng W, Chen S, Guo W, Tao M, Zhu L, Yang H (2002). A draft sequence of the rice genome (*Oryza sativa* L. ssp. indica). Science.

[4] Tuskan GA, Difazio S, Jansson S, Bohlmann J, Grigoriev I, Hellsten U, Putnam N, Ralph S, Rombauts S, Salamov A, Schein J, Sterck L, Aerts A, Bhalerao RR, Bhalerao RP, Blaudez D, Boerjan W, Brun A, Brunner A, Busov V, Camp-bell M, Carlson J, Chalot M, Chapman J, Chen GL, Cooper D, Coutinho PM, Couturier J, Covert S, Cronk Q, Cun-ningham R, Davis J, Degroeve S, Dejardin A, Depamphilis C, Detter J, Dirks B, Dubchak I, Duplessis S, Ehlting J, Ellis B, Gendler K, Goodstein D, Gribskov M, Grimwood J, Groover A, Gunter L, Hamberger B, Heinze B, Helariutta Y, Henrissat B, Holligan D, Holt R, Huang W, Islam-Faridi N, Jones S, Jones-Rhoades M, Jorgensen R, Joshi C, Kangasjarvi J, Karlsson J, Kelleher C, Kirkpatrick R, Kirst M, Kohler A, Kalluri U, Larimer F, Leebens-Mack J, Leple JC, Locascio P, Lou Y, Lucas S, Martin F, Montanini B, Napoli C, Nelson DR, Nelson C, Nieminen K, Nilsson O, Pereda V, Peter G, Philippe R, Pilate G, Poliakov A, Razumovskaya J, Richardson P, Rinaldi C, Ritland K, Rouze P, Ryaboy D, Schmutz J, Schrader J, Segerman B, Shin H, Siddiqui A, Sterky F, Terry A, Tsai CJ, Uberbacher E, Unneberg P, Vahala J, Wall K, Wessler S, Yang G, Yin T, Douglas C, Marra M, Sandberg G, Van de Peer Y, Rokhsar D (2006). The genome of black cottonwood, *Populus trichocarpa* (Torr. & Gray). Science.

[5] Jaillon O, Aury JM, Noel B, Policriti A, Clepet C, Casagrande A, Choisne N, Aubourg S, Vitulo N, Jubin C, Vezzi A, Legeai F, Hugueney P, Dasilva C, Horner D, Mica E, Jublot D, Poulain J, Bruyere C, Billault A, Segurens B, Gouyvenoux M, Ugarte E, Cattonaro F, Anthouard V, Vico V, Del Fabbro C, Alaux M, Di Gaspero G, Dumas V, Felice N, Paillard S, Juman I, Moroldo M, Scalabrin S, Canaguier A, Le Clainche I, Malacrida G, Durand E, Pesole G, Laucou V, Chatelet P, Merdinoglu D, Delledonne M, Pezzotti M, Lecharny A, Scarpelli C, Artiguenave F, Pe ME, Valle G, Morgante M, Caboche M, Adam-Blondon AF, Weissenbach J, Quetier F, Wincker P (2007). The grapevine genome sequence suggests ancestral hexaploidization in major angiosperm phyla. Nature.

[6] Sato S, Nakamura Y, Kaneko T, Asamizu E, Kato T, Nakao M, Sasamoto S, Watanabe A, Ono A, Kawashima K, Fujishiro T, Katoh M, Kohara M, Kishida Y, Minami C, Nakayama S, Nakazaki N, Shimizu Y, Shinpo S, Takahashi C, Wada T, Yamada M, Ohmido N, Hayashi M, Fukui K, Baba T, Nakamichi T, Mori H, Tabata S (2008). Genome structure of the legume, *Lotus japonicus*. DNA Res.

[7] Szczyglowski K, Stougaard J (2008). *Lotus* genome: pod of gold for legume research. Trends Plant Sci.

[8] Schmutz J, Cannon SB, Schlueter J, Ma J, Mitros T, Nelson W, Hyten DL, Song Q, Thelen JJ, Cheng J, Xu D, Hellsten U, May GD, Yu Y, Sakurai T, Umezawa T, Bhattacharyya MK, Sandhu D, Valliyodan B, Lindquist E, Peto M, Grant D, Shu S, Goodstein D, Barry K, Futrell-Griggs M, Abernathy B, Du J, Tian Z, Zhu L, Gill N, Joshi T, Libault M, Sethuraman A, Zhang X-C, Shinozaki K, Nguyen HT, Wing RA, Cregan P, Specht J, Grimwood J, Rokhsar D, Sta-cey G, Shoemaker RC, Jackson SA (2010). Genome sequence of the palaeopolyploid soybean. Nature.

[9] Cook DR (1999). *Medicago truncatula* - a model in the making. Curr. Opin. Plant Biol.

[10] Ane J-M, Zhu H, Frugoli J (2008). Recent advances in *Medicago truncatula genomics*. Int. J. Plant Genomics.

[11] Stougaard J (2001). Genetics and genomics of root symbiosis. Curr. Opin. Plant Biol.

[12] Udvardi MK, Tabata S, Parniske M, Stougaard J (2005). *Lotus japonicus*: legume research in the fast lane. Trends Plant Sci.

[13] Sato S, Tabata S (2006). *Lotus japonicus* as a platform for legume research. Curr. Opin. Plant Biol.

[14] Young ND, Udvardi M (2009). Translating *Medicago truncatula* genomics to crop legumes. Curr. Opin. Plant Biol.

[15] Desbrosses GG, Kopka J, Udvardi MK (2005). *Lotus japonicus* metabolic profiling. Development of gas chromatography-mass spectrometry resources for the study of plant-microbe interactions. Plant Physiol.

[16] Bonanomi G, Vinale F, Scala F (2009). The role of natural products in plant-microbe interactions. Plant-Derived Nat. Prod.

[17] Perry JA, Wang TL, Welham TJ, Gardner S, Pike JM, Yoshida S, Parniske M (2003). A TILLING reverse genetics tool and web-accessible collection of mutants of the legume *Lotus japonicus*. Plant Physiol.

[18] Wienkoop S, Saalbach G (2003). Proteome analysis. Novel proteins identified at the peribacteroid membrane from *Lotus japonicus* root nodules. Plant Physiol.

[19] Dam S, Laursen BS, Ørnfelt JH, Jochimsen B, Stærfeldt HH, Friis C, Nielsen K, Goffard N, Besenbacher S, Krusell L, Sato S, Tabata S, Thøgersen IB, Enghild JJ, Stougaard J (2009). The proteome of seed development in the model legume *Lotus japonicus*. Plant Physiol.

[20] Kato M, Miura A, Bender J, Jacobsen SE, Kakutani T (2003). Role of CG and non-CG methylation in immobilization of transposons in Arabidopsis. Curr. Biol.

[21] Schauser L, Roussis A, Stiller J, Stougaard J (1999). A plant regulator controlling development of symbiotic root nodules. Nature.

[22] Imaizumi R, Sato S, Kameya N, Nakamura I, Nakamura Y, Tabata S, Ayabe S, Aoki T (2005). Activation tagging approach in a model legume, *Lotus japonicus*. J. Plant Res.

[23] Sundaresan V (1996). Horizontal spread of transposon mutagenesis: new uses of old elements. Trends Plant Sci.

[24] Fukai E, Umehara Y, Sato S, Endo M, Kouchi H, Hayashi M, Stougaard J, Hirochika H (2010). Derepression of the plant chromovirus *LORE1* induces germline transposition in regenerated plants. PLoS Genet.

[25] Kumar A, Bennetzen JL (1999). Plant retrotransposons. Annu. Rev. Genet.

[26] Wessler SR (2006). Transposable elements and the evolution of eukaryotic genomes. Proc. Natl. Acad. Sci. USA.

[27] d'Erfurth I, Cosson V, Eschstruth A, Lucas H, Kondorosi A, Ratet P (2003). Efficient transposition of the *Tnt1* tobacco retrotransposon in the model legume *Medicago truncatula*. Plant J.

[28] Iantcheva A, Chabaud M, Cosson V, Barascud M, Schutz B, Primard-Brisset C, Durand P, Barker DG, Vlahova M, Ratet P (2009). Osmotic shock improves *Tnt1* transposition frequency in *Medicago truncatula* cv Jemalong during *in vitro* regeneration. Plant Cell Rep.

[29] Tadege M, Wen J, He J, Tu H, Kwak Y, Eschstruth A, Cayrel A, Endre G, Zhao PX, Chabaud M, Ratet P, Mysore KS (2008). Large-scale insertional mutagenesis using the *Tnt1* retrotransposon in the model legume *Medicago truncatula*. Plant J.

[30] Tadege M, Wang TL, Wen J, Ratet P, Mysore KS (2009). Mutagenesis and beyond! Tools for understanding legume biology. Plant Physiol.

[31] Benlloch R, d'Erfurth I, Ferrandiz C, Cosson V, Pío Beltrán J, Cañas LA, Kondorosi A, Madueño F, Ratet P (2006). Isolation of *mtpim* proves *Tnt1* a useful reverse genetics tool in *Medicago truncatula* and uncovers new aspects of *AP1*-like functions in legumes. Plant Physiol.

[32] Marsh JF, Rakocevic A, Mitra RM, Brocard L, Sun J, Eschstruth A, Long SR, Schultze M, Ratet P, Oldroyd GED (2007). *Medicago truncatula NIN* is essential for rhizobial-independent nodule organogenesis induced by autoactive calcium/calmodulin-dependent protein kinase. Plant Physiol.

[33] Wang H, Chen J, Wen J, Tadege M, Li G, Liu Y, Mysore KS, Ratet P, Chen R (2008). Control of compound leaf development by FLORICULA/LEAFY ortholog Single Leaflet1 (SGL1) in *Medicago truncatula*. Plant Physiol.

[34] Vassileva VN, Kouchi H, Ridge RW (2005). Microtubule dynamics in living root hairs: transient slowing by lipochitin oligosaccharide nodulation signals. Plant Cell.

[35] Vassileva VN, Fujii Y, Ridge RW (2005). Microtubule dynamics in plants. Plant Biotechnol.

[36] Vassileva V, Zehirov G, Ugrinova M (2010). Variable leaf epidermal morphology in *Tnt1* insertional mutants of the model legume *Medicago truncatula*. Biotechnol. Biotec.

[37] Ossowski S, Schwab R, Weigel D (2008). Gene silencing in plants using artificial microRNAs and other small RNAs. Plant J.

[38] Ni F-T, Chu L-Y, Shao H-B, Liu Z-H (2009). Gene expression and regulation of higher plants under soil water stress. Curr. Genomics.

[39] Shao H-B, Chu L-Y, Jaleel CA, Manivannan P, Panneerselvam R, Shao M-A (2009). Understanding water deficit stress-induced changes in the basic metabolism of higher plants - biotechnologically and sustainably improving agriculture and the ecoenvironment in arid regions of the globe. Crit. Rev. Biotechnol.

[40] Joosen RVL, Ligterink W, Hilhorst HWM, Keurentjes JJB (2009). Advances in genetical genomics of plants. Curr. Genomics.

[41] Sato S, Nakamura Y, Asamizu E, Isobe S, Tabata S (2007). Genome sequencing and genome resources in model legumes. Plant Physiol.

