# Supplementary material for: Recent Progress in Development of Tnt1 Functional Genomics Platform for Medicago truncatula and Lotus japonicus in Bulgaria
Source: Curr Genomics. 2011 Apr;12(2):147–52. doi: 10.2174/138920211795564313 (PMC3129049; doi:10.2174/138920211795564313)
Supplement: Supplementary file 1 [file CG-12-147_SD1.pdf]

SUPPLEMENTARY MATERIAL

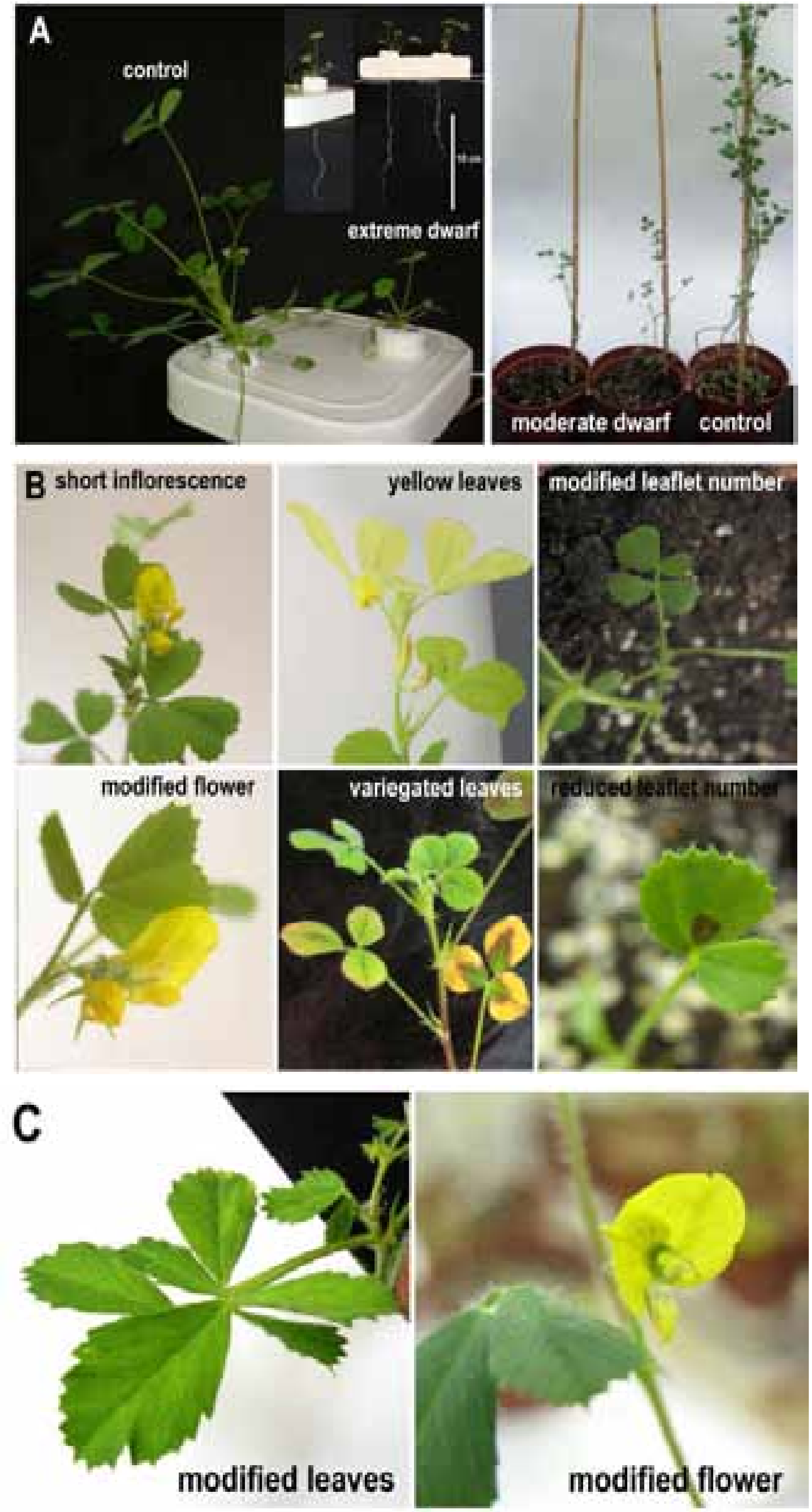

Suppl. Fig. (1). Visible phenotypic features of *Tnt1* mutant lines of *Medicago truncatula*. (**A**) extreme and moderate dwarf phenotype, (**B**) variable leaf and floral morphology, (**C**) *Tnt1* line with a strong leaf and floral phenotype.

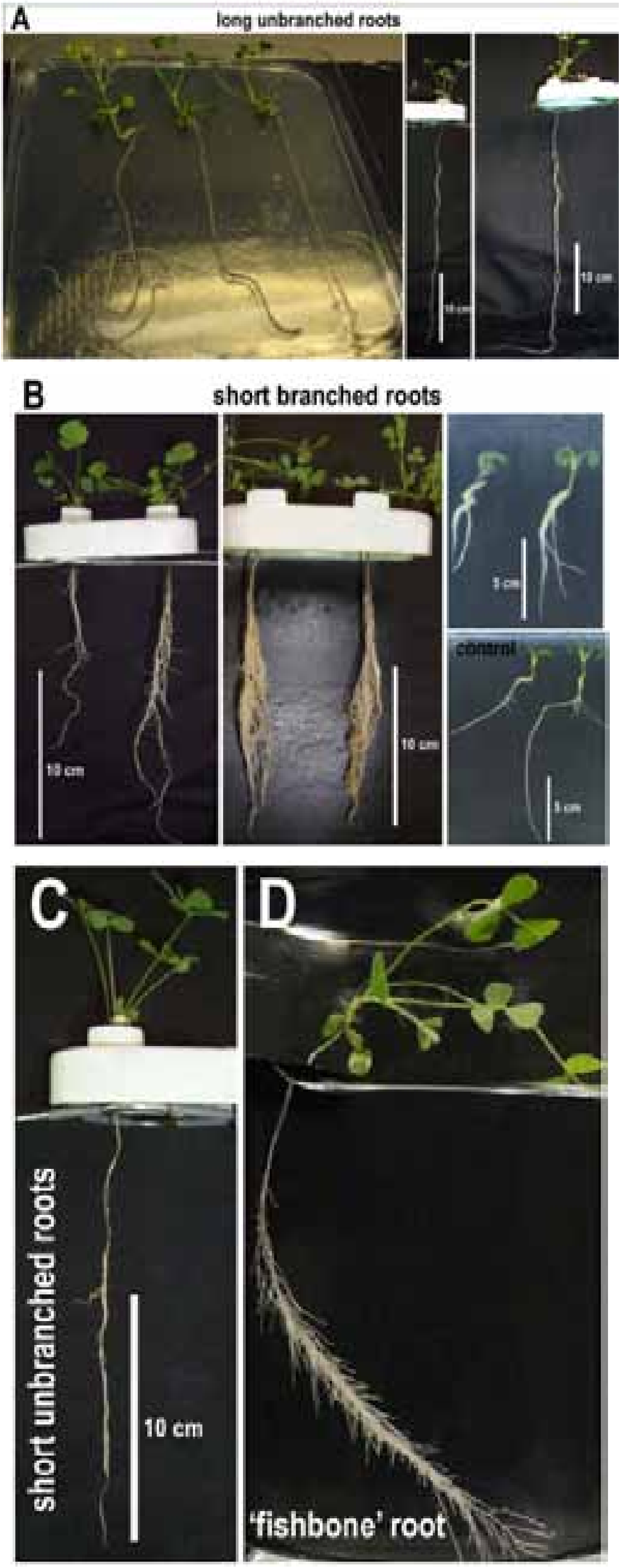

Suppl. Fig. (2). Major root phenotypes of *Tnt1* mutant lines of *Medicago truncatula*. (**A**) long unbranched roots, (**B**) short branched roots, (**C**) short unbranched roots, and (**D**) 'fishbone' root architecture.

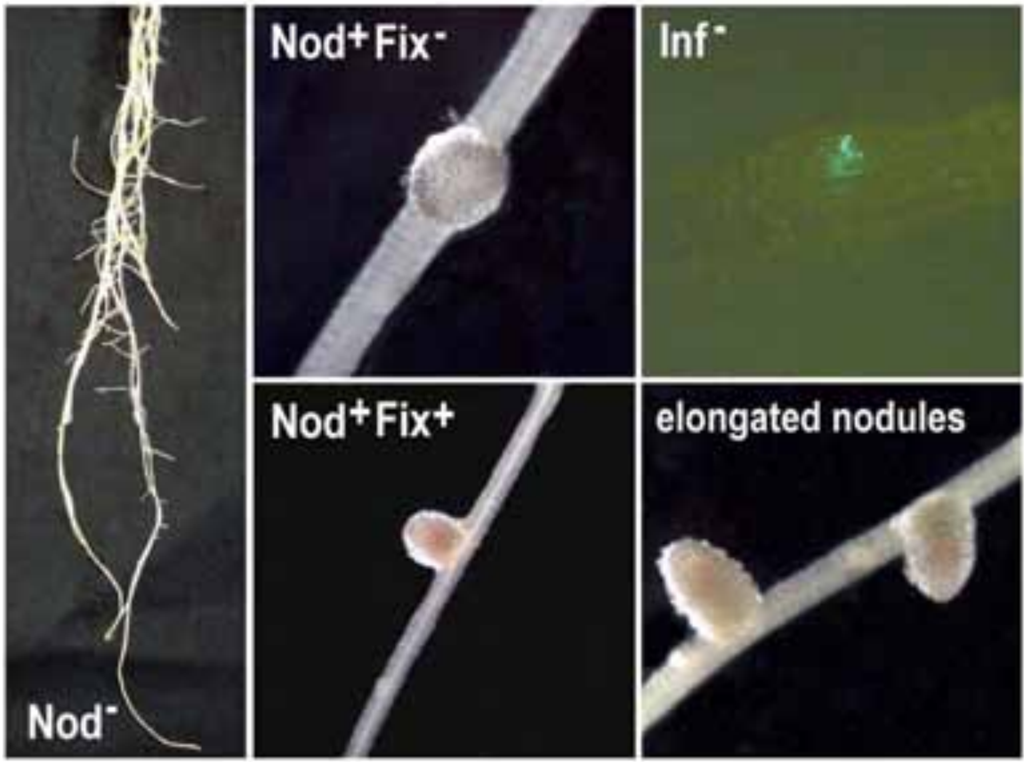

Suppl. Fig. (3). Variable symbiotic phenotypes of *Tnt1* mutant lines of *Medicago truncatula* - absence of nodulation (Nod^-), white (inefficient) nodules (Nod^-), efficient nodules (Nod^+), infection minus (Inf^-) mutants and elongated efficient nodules.
